# Miniplate Fixation to an Edentulous Mandibular Fracture in Panfacial Trauma

**DOI:** 10.7759/cureus.10562

**Published:** 2020-09-21

**Authors:** Dieter Brummund, Angela Chang, Joseph Michienzi

**Affiliations:** 1 Department of General Surgery, Aventura Hospital and Medical Center, Aventura, USA; 2 Department of Anesthesiology, Aventura Hospital and Medical Center, Aventura, USA; 3 Department of Oral Maxillofacial Surgery, Aventura Hospital and Medical Center, Aventura, USA

**Keywords:** mandible fracture, mental foramen, edentulous, panfacial trauma, assault, inferior alveolar nerve, 3-d miniplate, champy

## Abstract

A 70-year-old edentulous male presented with bilateral mandible and left midface fractures following an assault. Imaging confirmed fractures and showed mandible thickness greater than 20 millimeters. The patient was treated by open reduction internal fixation with miniplates via an intra-oral approach and recovered without deficit. While miniplate fixation and an intra-oral approach is typically reserved for the dentulous patient, this case illustrates that in select edentulous patients with sufficient bone thickness and amenable midface fractures this technique may be successfully utilized.

## Introduction

Mandibular fractures are the second most common fracture in facial trauma [1]. Mandibular fractures are classified according to location with the symphysis and body, angle, and condyle, each roughly accounting for one-third of all fractures. Due to force transduction, fractures often occur in multiple locations at points of weakness in the bone, such as the mental foramen, angle of mandible, and the thin bone of the condyles. Fractures can be horizontally or vertically favorable or unfavorable given their location. Favorability is determined by how the fracture line relates to the origin and insertion of the muscles of mastication due to the stabilizing or distracting forces they generate when firing.

Specific fracture patterns are unique to their respective traumatic etiology. Mandibular fractures have associated soft tissue injury up to 30% of the time and are associated with traumatic brain injury in up to 45% of cases and cervical spine injury in up to 9.7% of cases [2]. Particularly relevant to mandible fracture is the inferior alveolar nerve injury with prevalence in studies between 5% and 50%. Inferior alveolar nerve injury has been associated with isolated low- to medium-energy injury mechanisms such as assault, mandible fracture displacement, angle fractures, and sensory disturbance on preoperative exam [3].

Edentulism, by definition, is the lack of teeth. The edentulous population is more often older, frail, and suffering from pre-existing comorbidities and nutritional deficiencies. Edentulous mandible fractures are uncommon, occurring in 3% of all mandible fractures at some centers, and vary according to the regional prevalence of edentulism [4]. As described by Cawood et al. in his landmark anatomic study, a loss of alveolar bone and predictable changes in shape follows the loss of teeth [5]. Luhr et al. furthered the work of Cawood and developed his own system classifying the edentulous mandible by degrees of atrophy corresponding to mandibular height at its thinnest point with Class 1 (15-20mm), Class 2 (10-15mm), and Class 3 (<10mm) respectively [6]. Given the atrophic changes of the edentulous mandible, it is associated with a particularly high rate of malunion of 20% [7]. 

## Case presentation

**Figure 1:**
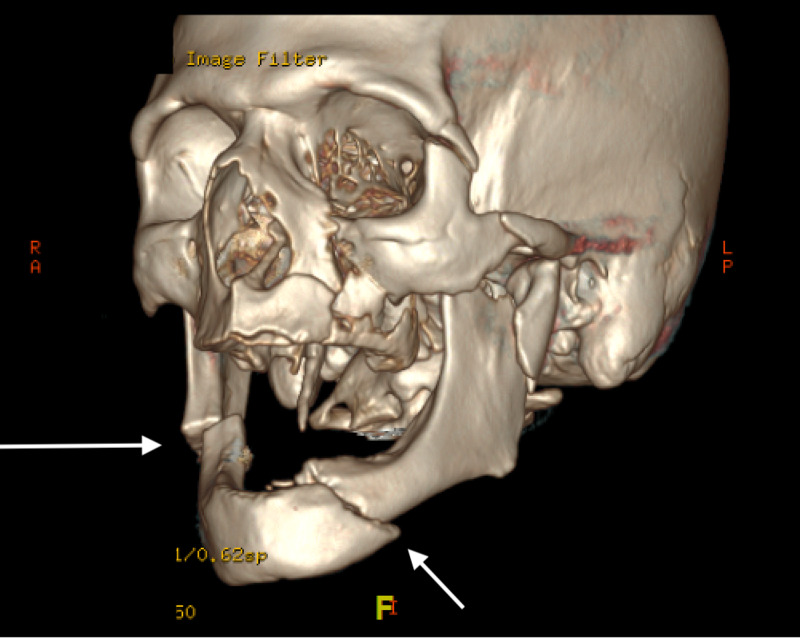
Displaced left parasymphaseal (short arrow) and right mandibular angle (long arrow) fractures Note involvement of left mental foramen

A 70-year-old edentulous man with a history of cocaine abuse, alcoholism, and homelessness presented with altered mental status, facial pain, and swelling after an assault. Physical examination revealed periorbital ecchymosis, intra-oral ecchymosis, and trismus. The patient was intubated in the trauma bay for airway protection, given his altered mentation. Laboratory findings were significant for moderate anemia with hemoglobin of 11.7 g/dL (reference range: 14-18 g/dL), a creatinine of 1.2 mg/dL (0.84-1.21 mg/dL) suggestive of mild dehydration, transaminitis with aspartate transferase of 82 u/L (7-55 u/L) and alanine transferase 48 u/L (8-48 u/L) consistent with history of alcohol abuse, and rhabdomyolysis with a creatine kinase level of 3229 u/L (55-170 u/L). Urine toxicology screen was positive for cocaine metabolites. Computed tomographic imaging of the brain and facial bones revealed panfacial fractures, including a horizontal unfavorable left mandibular fracture extending through the left mental foramen, a left condylar neck fracture with anterior subluxation of the temporal mandibular joint, a vertically unfavorable right mandibular angle fracture, a left lateral pterygoid plate fracture, comminuted impacted fractures of the left maxillary sinus involving the anterior lateral and superior walls, a displaced left zygomatic arch fracture, and a left greater wing of sphenoid fracture (Figure 1). Further imaging of the spine, chest, abdomen, and pelvis were negative for other acute traumatic pathology. The patient was extubated on post-injury day two and cleared for surgery.

**Figure 2:**
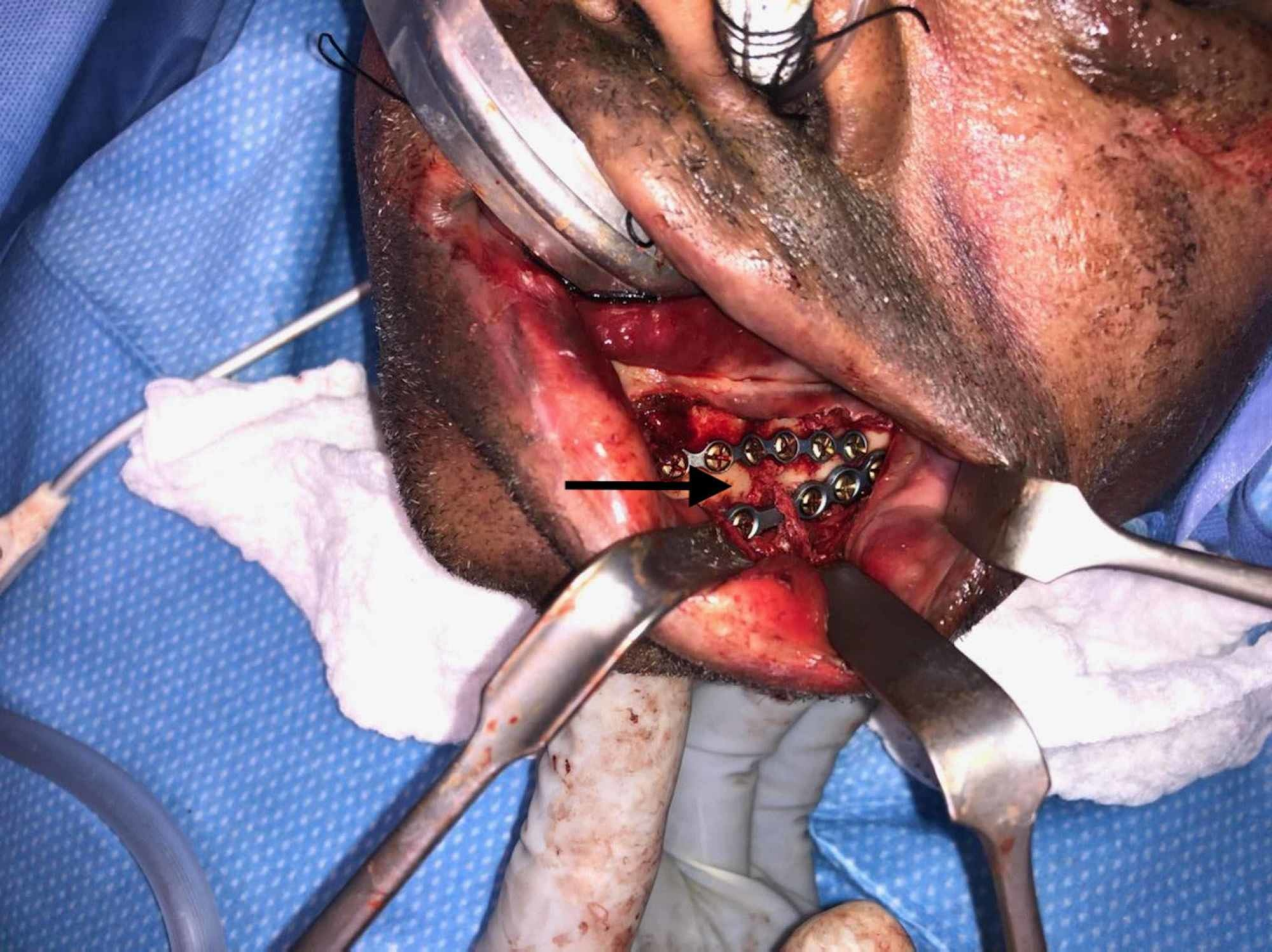
Intraoperative reduction of left parasymphaseal fracture with plating protecting inferior alveolar nerve (arrow)

**Figure 3:**
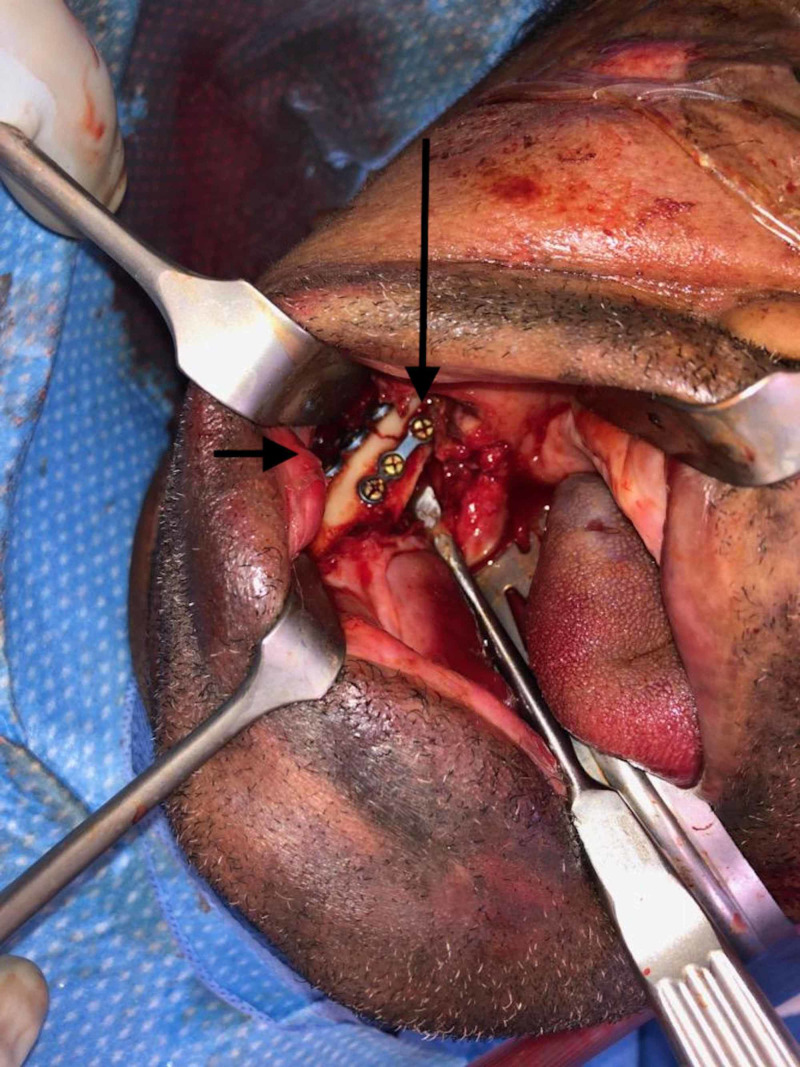
Right mandibular angle plating with vertical miniplate (long arrow) and horizontal 3D miniplate (short arrow)

On post-injury day three, the patient went to the operating room for open reduction and internal fixation with 2.3 mm miniplates using the Stryker Universal Mandible plating system (Stryker, Kalamazoo, USA). After nasotracheal intubation, the left parasymphaseal fracture was approached via an intra-oral incision with care to identify and protect the inferior alveolar nerve. The fracture was reduced with reduction clamp and then plated superiorly and inferiorly with two miniplates, bent so as to protect the inferior alveolar nerve from impingement, and fixated with mono-cortical screws (Figure 2). Thereafter the right mandibular body fracture was approached via an intra-oral incision and reduced with a reduction clamp. A miniplate was used superiorly to address the vertical buttress. Then using a transbuccal trocar, a three-dimensional miniplate was used to secure the horizontal buttress (Figure 3). Finally, the left lateral orbital wall fracture and left maxillary sinus fractures were reduced and plated in that order using a left intra-oral maxillary incision.

**Figure 4:**
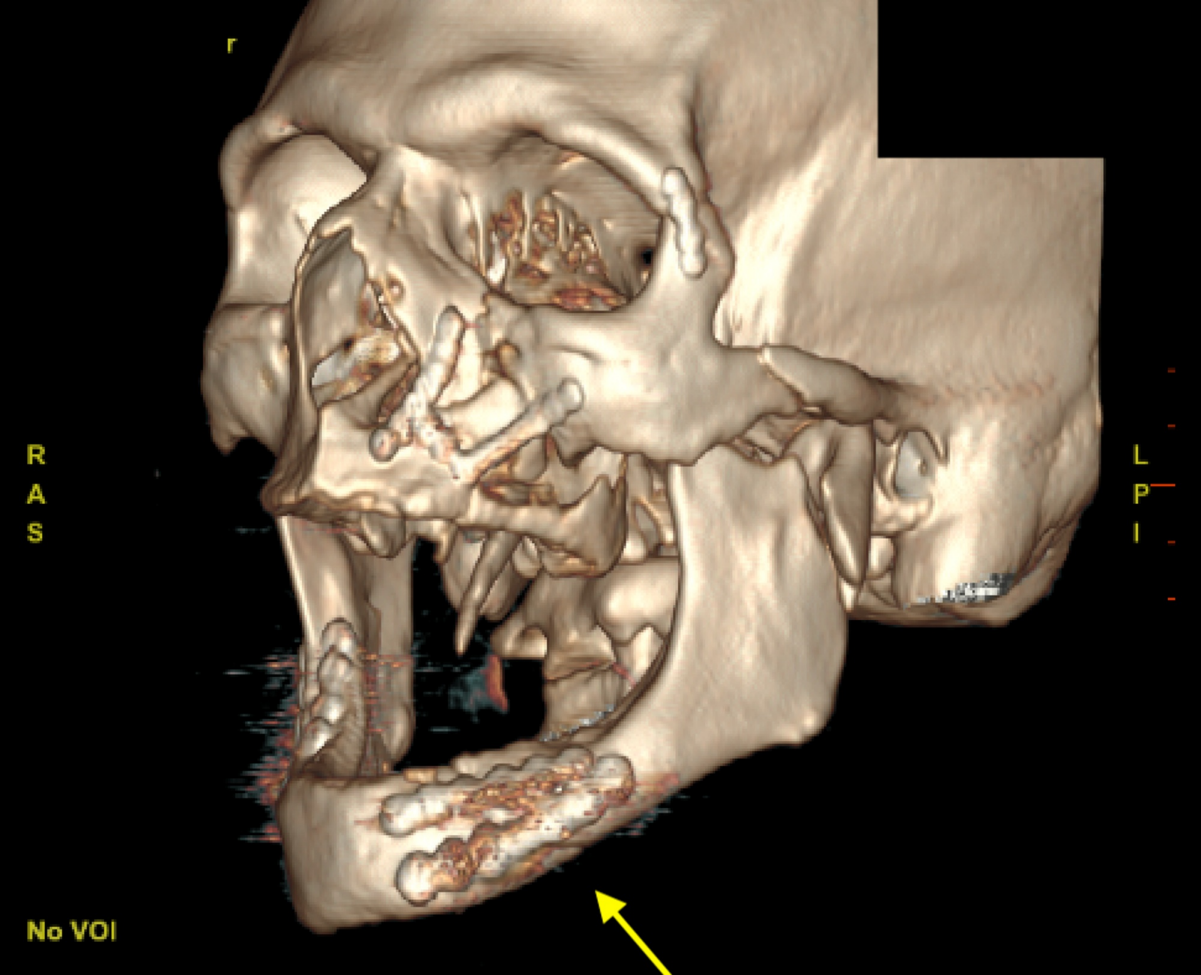
Postoperative reconstruction showing miniplate fixation surrounding left mental foramen

**Figure 5:**
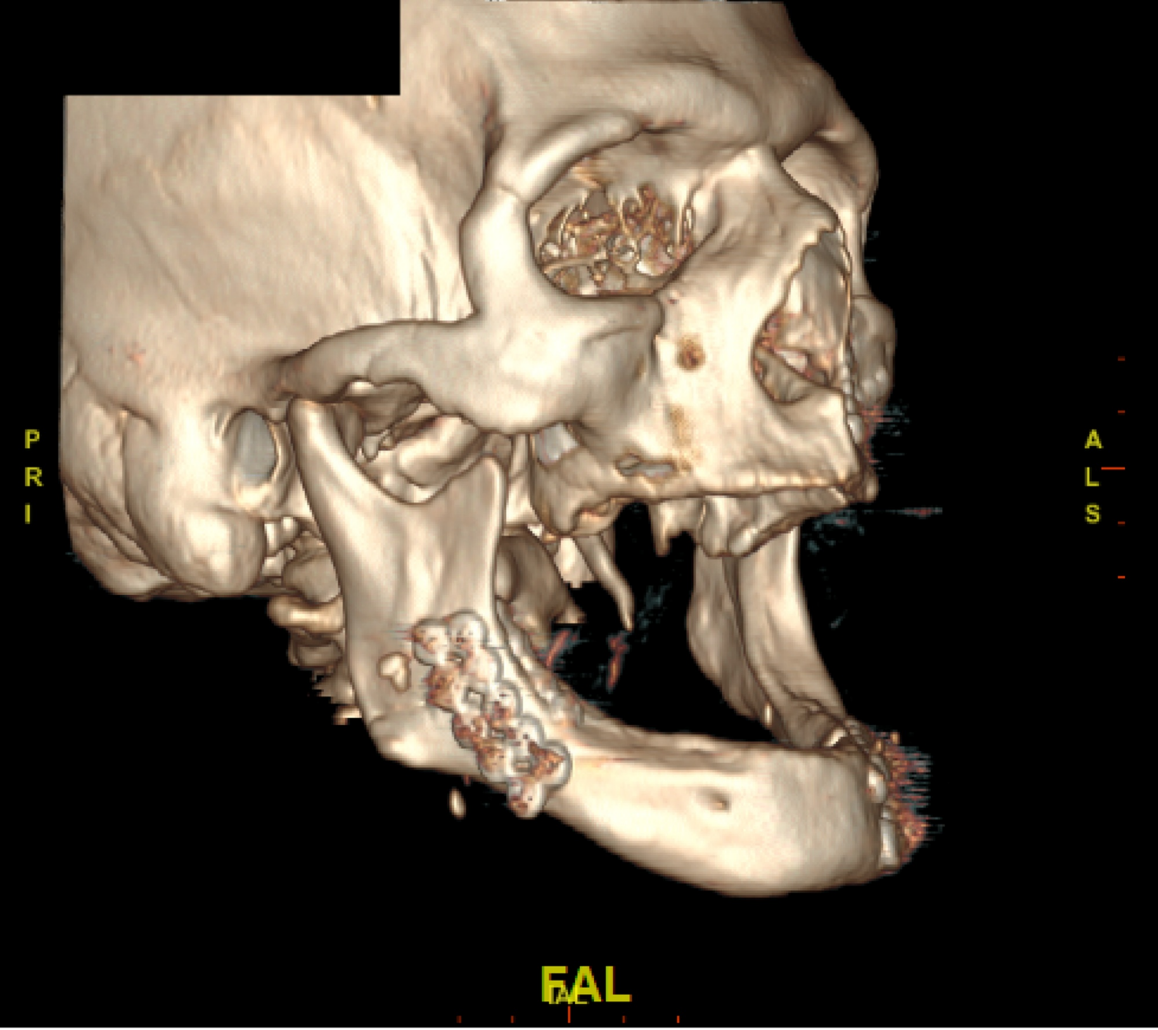
Postoperative reconstruction showing 3D miniplate fixation of right mandibular angle

The patient was successfully extubated postoperatively and started on a clear liquid diet overnight. Meticulous oral hygiene with chlorhexidine rinses and sinus precautions was initiated. On postoperative day one, he was advanced to a full liquid diet, and imaging was performed to confirm fixation. Imaging is notable for the miniplates that were shaped around the left mental foramen so as to not impinge on the inferior alveolar nerve (Figure 4). In addition, the three-dimensional miniplate spanning the right mandibular angle down to the inferior border allowed increased dispersion of masticatory forces to provide increased stability to the repair (Figure 5). The postoperative course was complicated by delirium tremens and substance withdrawal, successfully treated with benzodiazepines and dexometomidine drip in the intensive care unit. He was then transferred to the intermediate care unit on postoperative day three, at which time he was able to cooperate with a detailed cranial nerve exam and was found to have intact sensation in the distribution of the inferior alveolar nerve. He was discharged from the hospital on postoperative day five.

## Discussion

Closed reduction with wires and maxillomandibular fixation (MMF) was traditionally preferred for edentulous mandible fracture fixation, often by modifying the patient's own denture or using a Gunning splint with an anterior orifice allowing for oral intake. Given the lack of movement allowed with MMF, patients have more difficulty maintaining caloric needs and adequate pulmonary toilet. The edentulous geriatric patient with limited reserve may not be able to tolerate the six to eight weeks of fixation required for treatment [8].

Open reduction and internal fixation (ORIF) allows for an earlier return to function, but is technically more challenging to place on atrophic bone and can be complicated by non-union and plate extrusion. Open reduction of the mandible can be done via an intra-oral or extra-oral approach. The edentulous mandible is associated with atrophy of the alveolar and teeth-bearing regions, resulting in a more superficial inferior alveolar nerve and risking injury during the inciting trauma or intra-oral dissection. The extra-oral approach allows exposure of the mandible outside of the course of the inferior alveolar nerve, but care must be taken near the angle of the mandible as the marginal branch of the facial nerve may course up to 1-3 cm below in some patients.

Open reduction with load-carrying rigid fixation using thick reconstructive plates placed along the inferior border of the mandible takes the tension off the bone. Rigid fixation is primarily performed via an extra-oral approach. The submandibular Risdon incision is made 1-2 cm below the inferior border of the mandible and provides good exposure for angle or body fractures but only exposes one side. For symphysis fractures, a submental incision can be made. Finally, as described by Pereira et al., a transcervical incision provides exposure of the symphysis, angle, and body bilaterally in one incision and can be most advantageous in the geriatric edentulous patient where the incision can be hidden in the skin folds associated with aging [7]. 

Rigid fixation was the first open technique to be developed and is still preferred in atrophic edentulous mandible fractures due to its ability to withstand masticatory forces and provide stability required for healing. In the event of particularly atrophic bone in the fracture line, areas can be grafted with autologous cortical bone from the tibia or other donor sites [9].

Semi-rigid load sharing fixation utilizes miniplates placed along the buttresses of the mandible, which transfer the forces of mastication and facial expression, i.e., the alveolar and the inferior borders horizontally and the angle of the mandible vertically. Miniplates allow for an intra-oral approach. An incision is made 1 cm from the alveolar margin, providing a cuff of muscle, before carrying the dissection deeper to the periosteum. Care is taken to identify and isolate the inferior alveolar nerve, which courses along the groove in the lingual aspect of the mandible before exiting the mental foramen between the first and second premolars [10]. In the edentulous patient, preoperative review of imaging can facilitate surgical planning to locate the nerve foramina without the usual landmarks provided by the teeth.

Miniplates partially offload the bone while still allowing dynamic tension at the osteosynthesis line, stimulating bone healing. Due to their smaller size and low profile, miniplates require less periosteal dissection allowing for preservation of blood supply, less postoperative inflammation, and a shorter length of stay. In the elderly atrophic geriatric population, the stimulatory forces and preservation of blood supply may encourage bone healing. This approach has been successfully described by Mugino et al. in a case series of edentulous atrophic mandibular fractures [11]. Miniplate fixation can be a more technically demanding operation requiring increased precision and experience to place the plates in the smaller dissection planes. Problems can also occur with this approach when the underlying bone is critically atrophic as the miniplates may provide insufficient support.

Three-dimensional miniplate fixation is a new technique combining the benefits of semi-rigid fixation with those of rigid fixation. Mandibular angle fractures are at a particularly high risk of non-union and plate extrusion and are difficult to approach given their position. A transbuccal trocar allows for proper drill angulation. Alternatively, special 90-degree drills and drivers can be used to secure the hardware. The three-dimensional plate has found a niche in reducing these fractures due to two factors. Its shape allows for a distribution of load, thus providing greater stability than the traditional miniplate. In addition, its low profile allows for less periosteal stripping and easier placement by an intra-oral approach [12-13].

This case describes the use of miniplates using an intraoral approach to successfully reduce and plate mandibular fractures in the edentulous patient. A three-dimensional miniplate was used at the mandibular angle to provide additional support as this area is at high risk of complications, including plate extrusion and non-union. A benefit of the intra-oral approach was the ability to use another intra-oral incision to approach the midface fractures. Finally, due to the psychosocial dynamics of the patient, there was concern that he would be lost to follow up once discharged from the hospital, and thus miniplate fixation was chosen to allow for earlier return to function and shorter length of stay.

## Conclusions

This case report describes an intra-oral approach to open reduction and internal fixation with Stryker miniplates of mandibular body and angle fractures in an edentulous trauma patient. Open reduction internal fixation was chosen to allow for an earlier return to function and an intra-oral approach augmented by trans-buccal trocar. Care was taken during dissection to isolate the inferior alveolar nerve, and plates were shaped around it to protect it with the patient recovering without deficit. While not typically used for edentulous mandible fractures, a transoral approach and open reduction with miniplates was successful in this case due to the thickness of the patient's mandible and allowed for additional exposure and plating of the patient's midfacial fractures. This case highlights that each edentulous mandible fracture must be assessed on a case-by-case basis.
